# Deep Learning Approaches with Digital Mammography for Evaluating Breast Cancer Risk, a Narrative Review

**DOI:** 10.3390/tomography9030091

**Published:** 2023-06-06

**Authors:** Maham Siddique, Michael Liu, Phuong Duong, Sachin Jambawalikar, Richard Ha

**Affiliations:** Department of Radiology, Columbia University Medical Center, New York, NY 10032, USA; mas7101@nyp.org (M.S.);

**Keywords:** breast cancer risk, deep learning, convolutional neural network, digital mammography

## Abstract

Breast cancer remains the leading cause of cancer-related deaths in women worldwide. Current screening regimens and clinical breast cancer risk assessment models use risk factors such as demographics and patient history to guide policy and assess risk. Applications of artificial intelligence methods (AI) such as deep learning (DL) and convolutional neural networks (CNNs) to evaluate individual patient information and imaging showed promise as personalized risk models. We reviewed the current literature for studies related to deep learning and convolutional neural networks with digital mammography for assessing breast cancer risk. We discussed the literature and examined the ongoing and future applications of deep learning techniques in breast cancer risk modeling.

## 1. Introduction

Breast cancer remains the leading cause of cancer-related deaths in women worldwide [1], underscoring the vital importance of early detection and diagnostic screening for lesions or imaging phenotypes that may be indicative of cancer via screening modalities such as mammography. The current clinical models that dictate screening recommendations include established breast cancer risk factors such as later age at first birth, nulliparity, higher family income, and first-degree family history of breast cancer [2]. In addition, risk factors such as familial or genetic predisposition were also studied extensively. Mutations of the BRCA 1 and 2 genes, first documented in 1994 and 1995, account for 5–10% of breast cancer cases [3]. Familial and genetic predisposition accounts for 15% to 20% of diagnosed cases. While these risk factors explain such a significant proportion of diagnosed cases, the majority of breast cancer cases occur in women considered to be at average risk. Deep learning algorithms demonstrated effectiveness in various applications, including cancer detection and classification, making them valuable tools in identifying imaging biomarkers that may be indicative of breast cancer risk. When paired with a screening mammography exam, DL methods can calculate individual cancer risk separate from currently used clinical factors. In this review, our focus is to examine current studies utilizing deep learning techniques for breast cancer risk prediction using mammographic images.

Existing reviews in this domain included work from Acciavatti et al. [4], who comprehensively overviewed the utilization of DL methods across various imaging modalities from mammography and tomography to ultrasound and MRI, encapsulating the diverse landscape of risk modeling approaches in current practice. Similarly, Gastounioti et al. reviewed the application of DL methods applied to mammography in breast density evaluation and risk assessment [5]. We provide an updated analysis of the fast moving development of DL techniques for risk assessment. CNNs offer a promising avenue in this field due to their capability to handle high-dimensional data, thus making them ideal for the analysis of medical imaging data. Specifically, we review the application of CNNs for regressing continuous cancer risk scores from a standard mammographic study. Given the highly parameterized nature of CNNs and their requirement for substantial input data for training, mammography was chosen as the primary imaging modality. This decision stems from the substantial existing data repository of mammographic studies, which can facilitate the efficient training and performance assessment of CNNs. The purpose of this paper is to review the trends in scale, architecture, risk factors, and clinical factors that potentially influence the performance of CNNs in assessing breast cancer risk.

### 1.1. Screening Guidelines

Breast cancer screening guidelines are fundamental in determining a patient’s risk category as modeled by traditional risk factors. However, as Ren et al.’s systematic review pointed out, there is a significant variation in screening guidelines across different countries, even within the United States [6,7]. This inconsistency poses challenges to providing consistent patient care.

Personalized breast cancer risk assessment tools could help facilitate more effective screening guidelines, avoiding over-screening and unnecessary treatment while improving early cancer detection. High-risk patients may benefit from supplemental screening.

Individual risk assessment is possible with mammography, offering personalized imaging based on a patient’s unique characteristics. This shift from traditional population-level cancer risk models could improve the efficacy of screening guidelines. In the next section, we discuss traditional risk models and their influence on current screening recommendations, as well as potential improvements that can be made through advanced DL methods and individualized risk modeling.

### 1.2. Cancer Risk Models

Many breast cancer risk prediction models were developed over the past few decades. The Gail model, one of the first models proposed for breast cancer risk prediction, was extensively used and validated since it was developed in 1989 [8]. The Breast Cancer Surveillance Consortium (BCSC) and Tyrer–Cuzick models, which consider factors such as mammographic breast density, age, race/ethnicity, family history, and prior breast biopsies, were also used and validated to predict breast cancer risk [9].

The Gail, BCSC, and Tyrer–Cuzick breast cancer risk prediction models are commonly recommended for use in primary care settings. However, in clinical practice, these models often yield inconsistent results. Schonberg et al. found that the breast cancer risk estimates provided by these models poorly aligned with patient outcomes. Consequently, the use of these models resulted in inconsistent clinical recommendations, particularly for women in their 40s [10]. In a review conducted by Kim and Bahl in 2021, the performance of various risk prediction models was evaluated. The modified Gail/BCRAT models demonstrated an area under the receiver operator characteristic curve (AUC) of 0.58–0.74, BCSC models had an AUC of 0.61–0.67, and Tyrer–Cuzick models had an AUC of 0.71–0.75 [11].

### 1.3. Imaging Features for Risk Evaluation

The initial version of the Tyrer–Cuzick model did not account for imaging features such as breast density in cancer risk assessment. Women with over 50% mammographically dense breast tissue are at 3- to 5-fold greater risk for breast cancer compared to those with less than 25% dense breast tissue [12]. Around 64% of cancer diagnoses following routine screening mammography occur in women with dense breasts [13]. Risk prediction models that incorporate breast density demonstrate better performance than models relying solely on clinical factors. Tice et al. showed that models which incorporated measures of breast density were more effective at estimating the 5-year risk for invasive breast cancer [14].

Although the amount of breast density is a known risk factor, quantifying breast density is subjective and can vary widely amongst radiologists. Breast imaging reporting and data system (BI-RADS) was formed to standardize breast density assessment; however, visual assessment of mammographic density is prone to inter-and intra-reader variability. In addition, approximately half of the women in the US between the ages 40 and 74 years are classified as having dense breasts, making its use as a cancer risk factor less useful on an individual basis [15].

There is a need for a better and more reliable method of assessing breast cancer risk on an individual basis. Most current models provide risk estimates only at the population level [15]. An accurate assessment of a woman’s individual risk for developing breast cancer is necessary to guide personalized screening and prevention strategies. Women determined to be high risk can be offered more frequent surveillance and/or preventative measures such as surgery and chemoprevention therapy.

### 1.4. AI and Risk Assessment

**Figure 1:**
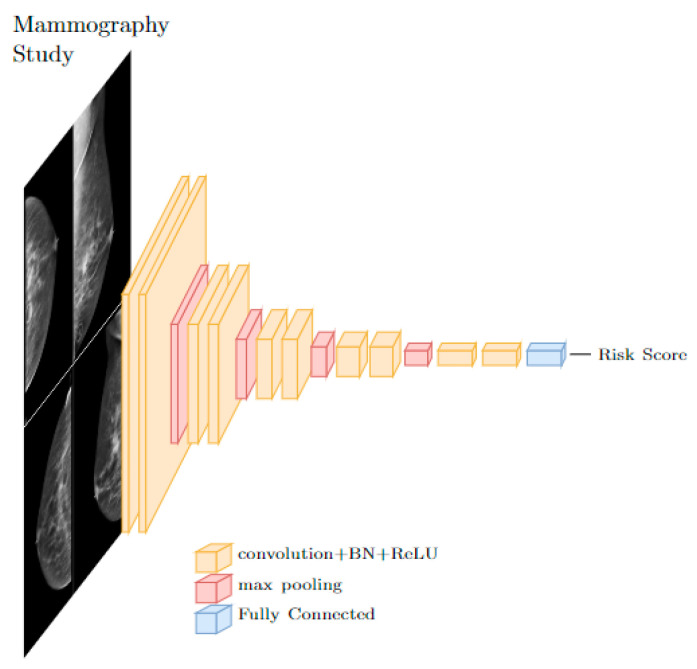
A typical CNN architecture for risk score prediction from images uses stacks of convolutional layers followed by fully connected layers.

Contemporary AI techniques involve data driven approaches, including deep learning (DL) and convolutional neural networks (CNNs). DL architectures consist of layers of interconnected and trainable neurons arranged into a network. The CNN, a DL architecture utilizing layers of convolution operations, is the most popular architecture for image-based models. These convolutional layers serve as image feature extractors, and when paired with fully connected neuron layers can be used for object detection, segmentation, and classification, to name a few [16,17,18,19]. CNNs can be applied to a patient’s mammography study with or without existing risk factors to calculate a patient specific breast cancer risk, an example architecture is depicted in Figure 1 below. This powerful technique was paired with many techniques within mammography including lesion detection and classification. We aimed to review the use of CNNs for a regression problem, that is, producing a continuous risk score from input of a normal mammographic study. Because CNNs are highly parameterized and require large amounts of input data to train, the mammographic study was chosen over other imaging modalities here because of the large existing base of data.

## 2. Methods

A search was conducted on 21 May 2023 with PubMed using the following keywords: Deep Learning, Convolutional Neural Networks, Mammography, and Breast Cancer Risk. Studies that discussed the utilization of deep learning techniques, such as artificial neural networks (ANN), convolutional neural networks (CNN), or transformer networks, for modeling short term breast cancer risk based on mammographic input were included in the review. In total, 78 studies were found, of which 51 were original studies. Studies that used DL methods for different tasks outside of breast cancer risk prediction, such as density assessment (13), lesion and microcalcification detection (4), classification (4), and segmentation (6) were excluded from the review. Articles not utilizing mammographic images (4) or utilizing mammography for tasks outside of cancer (1) were excluded. In total, 23 manuscripts were included in this review.

The number of examinations and the number of patients used in the training of the CNN model as well as the number of studies or patients that would later develop cancer were gathered when reported. CNN architectures, including the number of convolutional layers and specific techniques such as residual and dense connections, and inception and transformer methods were noted. All studies compared short term cancer risk (<5 years) or masking risk and not lifelong risk of cancer. Performance statistics for the most highly performing model from each study were aggregated and tabulated.

## 3. Study Selection

### 3.1. Small Scale Studies

Many small-scale studies involving fewer than 1000 cases also demonstrated the utility of CNNs for evaluating cancer risk. These studies tend to use a case–control cohort to train DL models, resulting in much smaller datasets upon which to train models. In these studies, short term cancer risk is assessed by analyzing images from normal mammograms prior to confirmed diagnosis.

Several studies studied short term risk by training CNNs to predict patients that would later be diagnosed with cancer. Arefan et al. trained a CNN-based short term cancer risk model with the prior normal mammogram exams from a 113-case cohort of 226 patients who underwent general population breast cancer screening [20]. A GoogLeNet-LDA CNN was employed to predict whether a patient would later develop breast cancer. The model achieved an AUC of 0.73 when using both MLO and CC views, outperforming the traditional imaging marker of percent breast density as a breast cancer risk predictor and showing reasonable performance for DL-based breast cancer risk marker. This study, however, needs validation from larger studies.

Kallenberg et al. [21], in 2018, also used CNNs to predict short term breast cancer risk from prior normal mammogram exams. They applied unsupervised deep learning to segment dense breasts, calculate breast density, and, subsequently, predict cancer risk on a dataset of 493 mammograms from healthy women in the Dutch breast cancer screening program and 668 mammograms from the Mayo mammography health study (MMHS) cohort. In total, 394 cancer cases and 1182 healthy control cases were used. A four-layer CNN-based model paired with a sparse autoencoder and softmax classifier was trained on 24 × 24-pixel patches. On validation, the risk model achieved an AUC of 0.57 (0.54–0.61). The performance was likely limited by low training numbers, shallow CNNs, and a risk model based on breast density.

Maghsoudia et al. [22] also used a dataset of 6368 normal mammographic exams from 414 women who later developed breast cancer an average of 4.7 years later and 1178 age matched controls to train a breast cancer risk assessment model. The CNN model was trained on bilateral CC and MLO images from the dataset to remove the pectoralis muscle and segment the dense vs. fatty tissue for breast density estimation from which cancer risk is calculated. This methodology, called Deep-LIBRA PD, yielded an AUC of 0.612 when paired with the four views of a screening mammography exam. Similar to the previous study, deep learning was used to calculate breast density, then used to model risk, meaning that the risk was predicted only by density.

Ha et al. also used prior normal screening mammograms from 737 of average risk women, 210 of which would later develop breast cancer. A CNN-based model, independent of the established measures of breast density, stratified breast cancer risk effectively [17]. Using a 21-layer CNN, the risk model was trained to predict the probability of later developing cancer. Overall, the model achieved an accuracy of 72% (95%CI, 69.8–74.4) in predicting patients who would develop breast cancer. In a follow up retrospective study with 23,467 consecutive patients, of which 121 would later develop cancer, the same CNN breast cancer risk model performed at an AUC of 0.654 compared to the 0.624 AUC of the breast cancer surveillance consortium model [23].

CNNs were also shown to be effective predictors of masking risk. Gastounioti et al. used contralateral mammogram studies from 106 women with unilateral invasive breast cancer and 318 age matched controls to train a CNN fused with traditional texture features to predict breast cancer risk [24]. The CNN architecture used 29 traditional texture features including gray level histogram, co-occurrence, run length, and structural features fused with two convolutional layers terminating in a logistic regressor. The model returned strong case–control classification performance with an AUC of 0.9 at a sensitivity of 0.81 and specificity of 0.98.

Li et al. [25], in 2017, showed the effectiveness of CNNs in evaluating both short term breast cancer risk as well as masking risk. The CNN model was trained on 456 mammography cases from 53 high-risk BRCA1/2 mutation patients, 75 high risk unilateral cancer patients, and 328 low risk patients. The CNN was compared to conventional computerized radiographic texture analysis (RTA). A fusion CNN-RTA classifier was also compared. BRCA1/2 versus low-risk discrimination performance was evaluated for area under the receiver operator characteristic curve (AUC) of 0.82 for RTA, ROC of 0.82 for CNN, and ROC 0.86 for fusion. Unilateral cancer prediction yielded AUC of 0.73 for RTA, AUC of 0.82 for CNN, and AUC of 0.84 for fusion in predicting unilateral cancer versus low risk. This indicates that CNN techniques performed at least at the same level as conventional techniques and, when fused, produced a better performance than each alone.

### 3.2. Towards Clinical Validation

Moving towards clinical validation, recent studies began to explore larger-scale datasets to assess the generalization of CNN-based models in breast cancer risk prediction. While earlier studies focused on smaller cohorts, more recent investigations involved substantial datasets consisting of thousands of cases from multiple institutions. These datasets were often assembled by sequential accession, better approximating a more representative cancer prevalence compared to smaller studies.

Wanders et al. used a patch-based three-layer CNN to model cancer risk using mammograms from a large cohort of 51,400 women, 898 of whom would be diagnosed with breast cancer within 2 years after their last mammogram, as indicated in the Netherlands Cancer Registry [26]. Cox proportional hazard analyses determined associations between texture pattern scores, volumetric density, and breast cancer risk. Discriminatory performance was evaluated using c-indices. CNN scores were positively associated with breast cancer risk (HR: 3.16, *p* < 0.001 for Q4 vs. Q1) with a c-index of 0.61. Classic imaging biomarkers as dense volume and percentage dense volume also showed positive associations with breast cancer risk (HR: 1.85 and HR: 2.17, respectively, *p* < 0.001 for Q4 vs. Q1). Fusion CNN and classic markers yielded c-index of 0.62 (*p* < 0.001). Deep-learning-based texture pattern scores on digital mammograms independently correlated with breast cancer risk, enhancing the ability to differentiate future breast cancer cases from non-cancer cases.

In 2019, Yala et al. used a CNN-based model trained with 88,994 screening mammograms from 39,571 women to model breast cancer risk [18]. The model utilized a CNN model fused with a risk-factor-based logistic regression model using traditional risk factors such as age, weight, height, menarche age, menopausal status, family history, BRCA status, history of atypia, and breast density. The model was compared against established breast cancer risk model that included breast density (Tyrer–Cuzick model, version 8). The image-only DL model showed an AUC of 0.68, RF-LR showed an AUC of 0.67, and TC showed an AUC of 0.62. The hybrid model achieved the highest AUC of 0.70. The study showed that a DL model that directly utilized the mammographic imaging data outperformed the clinical risk model Tyrer–Cuzick(TC) model (version 8). Lehman et al. [27] later compared this hybrid model with National Cancer Institute Breast Cancer Risk Assessment Tool (BCRAT) and TC on a dataset of 119,139 bilateral screening mammograms from 57,617 consecutive patients. The calculated AUC 0.68 of the deep learning model was higher than TC 0.57 and BCRAT 0.57 models. These studies showed the consistent outperformance of current clinical risk models by DL-based models.

Dembrower et al. also evaluated a CNN-based model to predict breast cancer risk [16] on 1466 mammogram studies from 278 women with breast cancer diagnosis and 12,568 studies from 2005 women with no known cancer. Each study in this dataset from Karolinska University Hospital, Sweden, consisted of a standard four-view full field digital mammographic study. Percent density and dense area metrics were calculated via LIBRA (UPenn) automated quantitative analysis. CNN-based 5-year risk scores for cancer development were modeled with the Inception Resnet V2 architecture using the 4-view mammographic images as well as age at acquisition, exposure, mA, breast thickness, and compression force. The non-image data were incorporated as auxiliary inputs of the Inception architectures during training. The DL model (AUC of 0.65) outperformed percent density (AUC of 0.57) and dense area (AUC of 0.60), while yielding the lowest false negative rate (FNR, 31%) compared to dense area (FNR, 36%) and percentage density (FNR, 39%). The study concluded that a DL model can more accurately predict which women are at risk for future breast cancer compared with traditional percent density measurements, with a lower false-negative rate, particularly for more aggressive cancers.

Zhu et al. (2021) used deep learning (DL) models to estimate the risk of interval and screening-detected breast cancers, considering clinical risk factors [28]. The study used 25,096 mammograms from 6369 women, of whom 1609 would develop screening-detected breast cancer and 351 would develop interval invasive breast cancer. The study compared the performance of a clinical risk factor model that, in part, utilized the radiologist reported breast imaging reporting and data system (BI-RADS) density with a CNN that utilized a DenseNet-121 architecture to calculate features for each view and average pooled features from all views to predict cancer risk. Comparing screening-detected cancer versus matched controls, the CNN model achieved a C-index of 0.66. The clinical model had a C-index of 0.62 and the combined CNN clinical model had a C-index of 0.66. When comparing patients with interval cancer versus controls, the CNN achieved a C-index of 0.64. The clinical model with BI-RADS density had a C-index of 0.71. The combined DL and clinical risk factors model yielded a C statistic of 0.72. The P values indicated that the DL model’s ability to detect screen and interval cancer was superior to the BI-RADS model (*p* = 0.99, *p* = 0.002, respectively), but inferior to the combined model (*p* = 0.03). The CNN outperformed the clinical risk factors model in determining screening-detected cancer risk. However, the CNN performed poorer for determining interval cancer risk compared to clinical risk factors.

In a follow up validation study published in 2022 by Yala et al., their DL risk model, Mirai, was tested on 128,793 mammograms from a globally diverse cohort of 62,185 patients [29]. Like the previous study, a standard mammographic exam as well as clinical risk factors were used to train the model. If the clinical risk factors were not available, the DL model would predict the risk based on only the mammographic data. Imaging, pathology results, and risk factors were collected from Massachusetts General Hospital, USA; Novant, USA; Emory, USA; Maccabi-Assuta, Israel; Karolinska, Sweden; Chang Gung Memorial Hospital, Taiwan; and Barretos, Brazil. The study validated the DL model performance in identifying high-risk patients across diverse cohorts. A sub analysis also showed similar performance of the DL model across a diverse racial population including African American and Caucasian patients. A concordance index (c-index), the generalization of AUC, showed the weighted performance of the predicted 1–5 year cancer risk.

A summary of the cohort sizes and performance metrics for the CNN-based breast cancer risk model studies are shown below in Table 1. Overall, the fusion techniques of CNNs and traditional features such as risk factor regression models and computed textures were the best performing models. The performance of CNN and clinical models started at parity and current CNN models consistently outperformed existing models. Due to the open source nature of the model used by Yala [18], its logical architecture and good performance, it is the most widely validated model to date.

### 3.3. Novel Applications of DL Models beyond Screening

Several studies showed novel applications of DL breast cancer risk models beyond screening. In 2021, Manley et al. designed a DL model to score risk [19]. Changes in risk score in women who underwent risk-reducing chemoprevention treatment such as Tamoxifen or Aromatase Inhibitors was evaluated. Of 541 patients in the study, 184 patients underwent chemoprevention treatment and 357 patients did not. The study showed that significantly more treated women decreased their breast cancer risk score compared to the controls. The score correlated negatively with chemoprevention treatment (*p* = 0.02). The study showed DL-based risk scores declined significantly with treatment. This methodology can be used to assess the efficacy of known chemoprevention agents as well as in testing new chemoprevention strategies.

In 2022, McGuinness et al. showed that DL-based risk models could be used to predict breast cancer relapse among women with operable hormone receptor (HR)-positive breast cancer [32]. In this retrospective study, the model was trained on data from women with stage I-III, HR-positive unilateral breast cancer diagnosed at their institution. Patients who received adjuvant endocrine therapy and had at least two mammograms (baseline, annual follow-up) of the contralateral unaffected breast were included in the study. Among the 848 women followed for a median of 59 months, there were 67 (7.9%) breast cancer relapses. A significant difference was observed in the mean absolute change in CNN risk score from baseline to 1-year follow-up between those who relapsed and those who remained in remission (0.001 vs. −0.022, *p* = 0.030). The study showed that short-term changes in the DL risk score in patients undergoing adjuvant endocrine therapy were associated with breast cancer-free interval and had potential to predict breast cancer relapse.

## 4. Discussion

### 4.1. Screening Implications

The use of CNN-based risk models in breast cancer screening holds implications for improving screening strategies. Supplementary screening was supported for intermediate and high-risk women that may benefit from additional follow up.

Breast cancer risk assessment is a critical component of comprehensive screening programs. It helps identify individuals who would benefit from early and supplemental screening, genetic testing, and preventive therapies, while aiding the general population in making informed screening decisions. Existing clinical algorithms, such as modified Gail/BCRAT, BCSC, and Tyrer–Cuzick exhibited varying performances. Their performances were measured by the area under the receiver operating characteristic curve (AUC), with values ranging from 0.57 to 0.82 [11].

Studies showed that deep learning-based models, incorporating traditional risk factors and mammographic images, can enhance existing epidemiology-based models. CNN models applied to datasets from MGH, Novant, Emory, Maccabi Assuta, Karolinska, CGMH, and Barretos reported C-index values ranging from 0.75 to 0.84 [29].

With approximately 39 million women undergoing mammograms annually in the United States, confusion persists among clinicians and patients regarding the optimal timing and frequency of screening. DL-based risk models emerged as potential tools to predict individual breast cancer risk and guide screening regimens. The agreement between CNN-validated algorithms and clinical models is at least on par, highlighting the potential of CNN-based models in enhancing breast cancer risk assessment and informing screening decisions.

### 4.2. Summary and Future Direction

Current screening and treatment guidelines assess risk from largely non-imaging risk factors such as patient demographics, family history of breast cancer, and genetic predisposition. Higher mammographic breast density is associated with higher risk, and a measurement of breast density is being incorporated into the latest clinical risk models such as the Tyrer–Cuzick model (version 8).

Convolutional neural networks (CNNs) demonstrated their effectiveness in addressing classification problems and were proven to be efficient image extractors for mammographic and other radiologic imaging biomarkers. One notable advantage of CNNs is their ability to be fused with established clinical risk factors such as hormone status and genetics. Extensive large-scale validation studies already indicated that CNN models perform with C-index values ranging from 0.75 to 0.84 [29] on large scale validation, which is on par with existing clinical risk models shown to perform with AUC between 0.57 and 0.82 [11], marking a significant achievement. However, there is still considerable room for improvement, suggesting the potential for further enhancements in CNN-based risk assessment models.

The 2022 validation study by Yala et al. [29] remains the largest of its kind, though it had some limitations. The model was trained on data from a single institution, sampling a limited patient population and homogenous clinical protocols. The model was also trained on mammograms from a single vendor. This necessitates validation on imaging from other vendors, as vendors use different anodes, filtration, and receptors in their acquisition. A retraining of the model with the combined datasets would likely improve performance and generalizability.

Beyond mammography, tomosynthesis is now widely available and routinely collected alongside 2D mammograms. CNNs will be able to utilize both the 3D and 2D imaging to further improve risk models. The power of CNNs scales with dimension, and tomosynthesis powered CNNs will eventually outperform 2D mammography powered CNNs. All models will need to be externally validated and prove generalizability before widespread adoption.

Overall, deep learning models outperformed the clinical models currently in use, though these models were found to be in the validation stages of development. With concordance indices averaging 0.78 [29], where an AUC of 0.7 or higher was generally considered acceptable for a risk prediction model to be useful, there is still much room for improvement and many new datasets to train with. The application of these models could help fine tune screening practices beyond traditional risk factors, which apply to broad populations and qualitative imaging characteristics.

## Figures and Tables

**Table 1:** Performance of studies utilizing CNN models in characterizing breast cancer risk from mammographic examinations. Parenthetical numbers indicate the number of examinations or patients that were diagnosed with breast cancer within 5 years. Only convolutional layers are counted for methods. Abbreviations: risk factors (RF), conventional texture analysis (CTA).

	Exams	Patients	Metric	Method
Arefan, 2020 [20]	226	226 (113)	0.73 AUC	8 Layer Inception CNN
Gastounioti, 2018 [24]	424	424 (106)	0.90 AUC	2 Layer CNN with CTA
Li, 2017 [25]	456	456 (75)	0.84 AUC	8 Layer CNN
Ha, 2019 [30]	1474	737 (210)	0.72 Accuracy	21 Layer Resnet CNN
Kallenberg, 2016 [21]	2069	2069 (394)	0.57 AUC	4 Layer CNN
Zhu, 2021 [28]	6369	6369 (278)	0.72 C-index	4 Layer CNN
Dembrowser, 2020 [16]	14,034	2283 (278)	0.65 AUC	Inceptionv2 CNN+RF
Michel, 2023 [23]		23,467 (121)	0.654 AUC	21 Layer Resnet CNN
McKinney, 2020 [31]		28,953 (1100)	0.889 AUC	DL model
Yala, 2019 [18]	88,994	39,571	0.70 AUC	19 Layer Resnet CNN with transformers +RF
Wanders, 2018 [26]	51,400 (898)	51,400 (898)	0.61 C-index	3 Layer CNN
Lehman, 2022 [27]	119,139	57,635	0.68 AUC	19 Layer Resnet CNN with transformers +RF
Yala, 2022 [29]				19 Layer Resnet CNN with transformers +RF
MGH	25,855 (588)	7005 (233)	0.75 C-index	
Novant	14,157 (235)	5887 (123)	0.75 C-index	
Emory	44,008 (1003)	16,495 (495)	0.77 C-index	
Maccabi Assuta	6187 (186)	6189 (186)	0.77 C-index	
Karolinska	19,328 (1413)	7353 (799)	0.81 C-index	
CGMH	13,356 (244)	13,356 (244)	0.79 C-index	
Barretos	5900 (146)	5900 (146)	0.84 C-index	

## Data Availability

Not applicable.
